# Linkages of quality of life with falls and injuries among older people in India

**DOI:** 10.1038/s41598-025-29486-1

**Published:** 2025-11-24

**Authors:** Anil Kumar Pal, Sanjay Kumar Pal

**Affiliations:** 1 Department of Family & Generations, International Institute for Population Sciences, Mumbai, 400088 India; 2 Department of Fertility & Social Demography, International Institute for Population Sciences, Mumbai, 400088 India; 3 International Institute for Population Sciences, Mumbai, 400088 India

**Keywords:** Quality of life, Falls, Injuries, Older people, India, Health care, Geriatrics, Public health, Quality of life

## Abstract

Falls and injuries and their consequences impact people’s quality of life (QoL), especially in older people. This study assesses the level of falls, injuries, and QoL and examines the linkages of QoL with falls, and injuries among older people in India. The study used data from the Longitudinal Ageing Study in India, Wave-I (2017-18). Univariate, bivariate, and ordered logistic regression analyses were performed for analysis. The study reveals that 13% and 14% of older adults experienced major falls and injuries, with 40% experienced low and 30% each experienced medium, and high QoL. The results suggest that older people with no self-care had higher major falls (21.7%) and injuries (22.6%), while those who could not perform their usual activities had higher major falls (17.3%) and injuries (18.4%). Ordered logistic regression results showed that older people with minor and major falls were 30% and 39%, respectively, less likely to have higher QoL. Older people with injuries were 35% less likely to have higher QoL. This study’s findings suggest that falls and injuries were crucial factors in reducing the QoL of older people. The findings indicate the importance of improving the QoL of older people by reducing the risk of experiencing falls and injuries.

**Supplementary Information:**

The online version contains supplementary material available at 10.1038/s41598-025-29486-1.

## Introduction

The global population is ageing rapidly, with the most significant increases in low- and middle-income countries (LMICs) due to demographic changes and medical advances. Projections show that the number of older people aged 60 and above will double by 2050 from 1 billion in 2019 to 2.1 billion by 2050, with more than 80% of the population residing in LMICs^1^. According to the Indian population census 2011, India, which recorded more than one billion population, made it the second most populous country in the world but now India, with more than 1.4 billion population, is the most populous country in the world^2^. Among them, approximately 103 million adults who were aged above 60 years with 8.6% of the total population. Projections suggest that the share of older population (60 and above) will rise to 19.5% (319 million) by 2050^3^.

Older adults in India face numerous health challenges, with falls being among the most significant. As defined by the World Health Organization (WHO), falls include slips or trips that result in an unintentional descent to the ground or a lower level^4^. Falls are the leading cause of injury, disability, and early death in adults over the age of 65. As the global population ages rapidly, preserving the health and independence of older adults is increasingly vital^5^. Over one-third of adults aged 65 and above fall each year, with more than half experience repeated falls, making falls a leading cause of death in developed countries^6^. Although, people of all ages can fall, but older adults have been found to be particularly at risk due to musculoskeletal changes and joint weakness that increase their risk of fractures^7^.

Falls and related injuries pose a major public health concerns among India’s older population, significantly affecting their quality of life (QoL). A systematic review reported that 65.6% of older adults with falls sustained injuries, including lower limb injuries (34.4%), cuts (38%), fractures (12.5%), and disabilities (11%)^8^. Data from Longitudinal Ageing Study of India (LASI) indicate that visual impairment is a key risk factor, which showed a 16% increased fall risk for those with low vision and 40% for those with blindness, with women being higher vulnerability^9^. Frailty also contributes notably, with frail individuals experiencing more falls (15.4% vs. 11.9%) and injuries (6.7% vs. 5.3%) as compared to their non-frail counterparts^10^.

Environmental factors such as poor lighting, unsafe public transport, and hazardous surroundings further elevate fall risks and limit social participation^11,12^. Falls and injuries pose a major socioeconomic challenge for older adults living alone, particularly those in single-family homes with poorer financial conditions and social support, which restrict their access to follow-up care^13^. In addition to injuries, falls often result in developed post-fall anxiety, reduced physical activity, and disrupted daily routines, increase social isolation, depression, and diminished well-being^14–22^.

Findings from several studies indicate that physiological and behavioural risk factors for falls include problems with gait, balance, strength, and vision, joint weakness, and excessive alcohol use^23–25^. The Centres for Disease Control and Prevention reports revealed that among older people, falls are one of the leading causes of death and disability; in this age group, falls cause 20% to 30% of injuries and 50% of injury-related hospitalizations^26^. Previous studies have reported the prevalence of falls among older adults in India to range from 26% to 37%, with a pooled prevalence of approximately 31% (95% CI: 23–39)^27,28^. Even when falls may not cause major injuries, they still affect older people’s mobility and functionality. Approximately one-third of persons aged 65 years and above have fallen at least once every year^29–31^. An Indian study also identified key risk factors for falls in this population, emphasizing the role of sociodemographic characteristics, environmental conditions, underlying health problems, and medical interventions^8^. Hospital stays for fall-related injuries often range from 4 to 15 days and are longer for cases involving fragility, hip fractures, or advanced age. Additionally, over 30% of older adults limit daily activities, and 30% to 55% report a fear of falling^32^. Falls and injuries in older adults reduce autonomy, increase dependency and fear, and lower QoL. Those who fall often have more comorbidities and require medical attention^33^.

Contrary to the need to maintain a balanced diet to strengthen bones and prevent trauma from injuries, the majority of older adults have inadequate nutrition^34^ In later life, older adults face challenges like multimorbidity, poor mental and functional health, insecurity, and social exclusion. Researchers increasingly advocate for prioritizing QoL over longevity through targeted funding and studies^35^. While all age groups are at risk of falls, older adults are more vulnerable due to musculoskeletal decline, leading to higher chances of fractures, increased healthcare needs, and post-fall syndrome that limits mobility and daily functioning. Numerous types of research have identified risk factors for falling; however, most of these studies looked at the older population in local areas rather than focusing primarily on older people who live alone^36^.

Recent studies also highlight that falls and fear of falling are directly related to health-related quality of life (HRQOL), especially among older people^15,37^. Other studies suggest that those with mobility issues and dependency with others for daily routine work are more prone to fall and tend to have lower HRQOL compared to independent older people^38–40^. Furthermore, studies suggest that HRQOL and falls are interconnected, with those experiencing falls often reporting reduced subjective well-being^41–43^.

However, most of the prior research originates from higher-income countries (HICs) with only some studies from LMICs and Indian community-based studies only included a small number of subjects or perhaps only a particular community. Therefore, the research on the link between HRQOL with fall and injuries among older persons requires an India-level study with representative samples. In order to obtain a more thorough and integrated understanding of falls and injuries and their contributing factors in the older population, HRQOL was measured using the Euro-QOL five-dimension questionnaire (EQ-5D), the visual analog scale (VAS), and chronic disease. With this backdrop, the present study assesses the level of falls, injuries, and QoL and examines the linkages of QoL with falls, and injuries among older people in India.

## Methods and materials

### Data source

This study uses data came from the first Wave of Longitudinal Ageing Study in India (LASI). In order to examine the health, economic, and social factors that influence population aging, LASI conducted a comprehensive nationwide survey in 2017-18. The LASI, Wave-1 covered a panel sample of 72,250 individuals age 45 and above and their spouses, including 31,464 elderly persons age 60 and above and 6749 oldest-old persons age 75 and above from 35 states and union territories (UTs) of India (excluding Sikkim). LASI used a multistage stratified area probability cluster sampling methodology to arrive at the final units of observation. This included older individuals aged 45 and up and their spouses of any age.

The survey employed a four-stage sampling design in urban areas and a three-stage sample design in rural areas. Choosing Primary Sampling Units (PSUs) and sub-districts (Tehsils/Talukas) was the first step in each state or UT. The second step was choosing villages in rural areas and wards in urban areas within the selected PSUs. In the third round, households were selected from designated communities in rural areas. On the other hand, sampling in metropolitan areas requires an extra step. In the third stage, a Census Enumeration Block (CEB) was chosen randomly in each urban area. Households from this CEB were selected in the fourth stage.

For the present study, a sample of 31,464 individuals aged 60 and above years was used to ensure a robust and representative analysis of health and QoL outcomes among the older adults.

## Methods

### Outcome variables

The outcome variable for this study is a health-related quality of life (HRQOL), which is interchangeably used as a quality of life (QoL). HRQOL is defined as a multidimensional construct encompassing the perceived impact of health status or diseases on an individual’s physical, mental, and social functioning. This indicator serves as a comprehensive indicator of the extent to which health influences overall well-being and daily life activities^44,45^. We assessed the HRQOL of each subject based on their responses to the Euro-QOL five dimensions (EQ-5D) questionnaire, which may suffice to compute HRQOL based on EQ-5D, a generic measure of HRQOL. The EQ-5D comprises five dimensions (mobility, self-care/activities of daily living (ADL), usual activities/instrumental activities of daily living (IADL), pain, and depression-CES), and each dimension is categorized into two levels of indicating presence or absence (no and yes)^46^. The EQ-5D questionnaire is currently available in two formats: EQ-5D-3 L and EQ-5D-5 L, where “L” denotes the number of response levels used to assess health status across five dimensions. The present study employed the EQ-5D instrument with two levels of health status measurement across all five dimensions^47^. The five health dimensions captured in the LASI dataset are described below.

The first dimension of EQ-5D measurement is defined as mobility. Respondents were asked mobility-related nine questions in LASI such as walking 100 yards, sitting for two hours or longer, rising from a chair after sitting for a long time, climbing one flight of stairs without stopping, stooping, kneeling, or crouching, reaching or extending arms above shoulder level (either arm), pushing or pulling heavy objects, lifting or carrying weights over five kilograms, such as a heavy grocery bag, picking up a coin from a table, etc. All mobility-related conditions were recoded as 1 “yes” (having difficulty) and 0 “No” (no difficulty).

The second dimension of EQ-5D is described as self-care/ADL. ADLs are widely accepted as a core measure of self-care, particularly in ageing research and clinical practice. ADLs were measured using the Kartz Index of Independence, which encompass basic tasks such as bathing, dressing, eating, toileting, getting in or out of bed and mobility functions that are essential for maintaining independence and well-being in older adults. Each item (ht401-ht406) was coded independent = 1 and dependent = 0, summed to a score of 0–6 (6 = full independence; 0 = complete dependence). The composite was dichotomized as ADL limitation (0–2) vs. no limitation (3–6), with missing values set to missing. The Katz Index is widely applied in geriatric and epidemiological studies to assess functional ability and disability^48^. Impairments in ADL reflect diminished functional capacity and are predictive of adverse outcomes, including hospitalization, institutionalization, and mortality. A recent study continue to validate the use of ADL as a robust indicator of functional health or self-care^49^. ADL difficulty was categorized as “no” (0–2 = 0)” or “yes” (3–6 = 1).

For the third dimension of EQ-5D “no” and “yes” were used to code difficulty in instrumental activities of daily living (IADL). IADLs are not always related to a person’s basic functioning but allows them to live independently in a community. IADLs serves as a critical measure of *usual activities* in older adults, as they encompass tasks essential for independent living within the community if they were experiencing any issues that would persist for longer than three months, such as cooking a hot meal, going grocery shopping, making a phone call, taking medication, cleaning or gardening, handling money (paying bills and keeping track of expenses), or navigating unfamiliar areas or locating an address. Unlike basic ADLs, IADLs reflect an individual’s ability to interact with their environment and maintain social roles, making them highly relevant to assessing usual daily functioning. Limitations in IADLs are often early indicators of cognitive or physical decline and are strongly associated with reduced QoL and increased care needs^50^.

The fourth dimension of EQ-5D is the pain, which was coded as “no” and “yes.” Pain-related questions were asked to the respondent in LASI, “Are you often troubled with pain”?

Furthermore, the fifth dimension of the EQ-5D measurable variable is depression. Depression-related questions were asked of the respondents to measure the depression symptoms by using the CES-D (Centre for Epidemiological Studies-Depression) scale^51^. A total of 10 questions were included in this study to assess depression, covering aspects such as trouble concentrating, feeling depressed, fatigue, fearfulness, loneliness, irritability, the sense that everything requires effort, hopelessness, happiness, and overall life satisfaction. These items were converted into binary outcome variables, combined to yield a total score ranging from 0 to 10. Based on the scoring, a total score of 0 to 3 was classified as “not depressed,” while a score of 4 to 10 was categorized as “depressed.”

Principal Component Analysis (PCA) was employed to derive a composite variable by using all five dimensions with binary nature variables, with scores ranging from 0 to 0.79. Using the ‘xtile’ command, this variable was categorized into three groups based on their weighted score ranges. These categories were subsequently recoded as low QoL, medium QoL, and high QoL.

### Exposure variable

Falls and injuries were assessed as exposure variables using a set of three questions designed to determine the extent and nature of the fall experienced by the individual. Have you fallen in the last two years? “Participants were asked at the start.” “How many times have you fallen in the last two years?” and “Did you injure yourself sufficiently in that fall (or any of those falls) to require medical treatment?” The falls were categorized into three categories no falls, minor falls, and major falls. *Injuries* were defined as falls that required medical attention. “In the past two years, have you sustained any major injuries?” with a “yes” or “no” response. Moreover, the survey included causes of fall injuries based on the question, “What was the cause of that injury?” Accidents in traffic, being struck by a person or object, fire, flames, burns, electric shock, drowning, poisoning, animal attack or bite, fall, and other responses.

### Independent variable

The independent variables of this study were age group, sex, place of residence, level of education, marital status, regions, castes, living arrangement, work status, and monthly per capita expenditure (MPCE) quintile and multimorbidity. Multimorbidity was categorized into three categories. If an individual has no morbidity defined as “No morbidity,” individuals with 1 morbidity defined as “1 morbidity” and individuals with 2 or more morbidity categorised as “2 or more morbidity”.

### Inclusion/exclusion criteria

This study used the secondary data from the Longitudinal Ageing Study in India (LASI), Wave-I (2017-18). The dataset is nationally representative and include information on individuals aged 45 years and above.

#### Inclusion criteria

In the study sample, we included older people age 60 and above with 31,464 sample of older people out of 72,250 people in India excluding Sikkim, have complete information on variables related to falls, injuries, QoL and other socio-demographic characteristics.

#### Exclusion criteria

In this study individuals younger than 60 years were excluded, resulting in a total sample of 40,786 older people excluded.

### Sample size of the study

After applying the inclusion and exclusion criteria, a total of 31,464 samples of older people were included in the final analysis. Since the study is based on secondary data, no additional sample size calculation was required; instead, we used the available sample from the Individual dataset after cleaning and filtering.

### Statistical analysis

The study participants’ general characteristics and distribution were determined using descriptive analysis. The QoL is the outcome of our interest. Furthermore, bivariate analysis has been used to see level of the QoL, falls, and injuries with socioeconomic characteristics of women. The Chi-square test was used to determine if there is a significant association between the socio-demographic characteristics and the QoL. The p-value is a measure to determine whether the relationship between the socio-demographic characteristics and QoL is statistically significant. The principal component analysis (PCA) was used to create the health-related quality of life (HRQOL) index and make a score to create a variable with QoL.

The findings were also carved out using ordered logistic regression analysis. The QoL were categorized as “Low QoL,” “Medium QoL,” and “High QoL.” The ordered logistic regression model is a regression model for a response variable with an ordinal value. The model is based on the response variable’s “cumulative probability.”

An ordered logistic regression model can be written as follows:

$$\:Logit\:\left(pi\right)=ln\left(\frac{\mathrm{p}\mathrm{i}}{1-pi}\right)={\beta\:}_{0}+{{\upbeta\:}}_{1}{\mathrm{x}}_{1}+{{\upbeta\:}}_{2}\:{\mathrm{x}}_{2}+\dots\:+{{\upbeta\:}}_{k}\:{\mathrm{x}}_{k}$$

Where $\:{\beta\:}_{0}\:$is the intercept, $\:{{\upbeta\:}}_{1},\:{{\upbeta\:}}_{2},\dots\:.\dots\:\dots\:,{{\upbeta\:}}_{k}\:$are the regression coefficients indicating the relative effect of a particular explanatory variable on the outcome, $\:{\mathrm{x}}_{1},\:{\mathrm{x}}_{2},\dots\:.\dots\:\dots\:,{\mathrm{x}}_{k}$, were the control variables^52^. A confidence interval (CI) is a range of values, derived from sample data, that is used to estimate an unknown population parameter, with a specified level of confidence (e.g., 95%), indicating the degree of uncertainty or precision of the estimate.

In Table 5, Model I presented the null model, which indicated the association between QoL and falls. Model II presented the adjusted odds ratio for the QoL of older adults after controlling for all the selected independent variables related to falls. Subsequently, Model III presented the null model assessing the relationship between QoL and injuries among the elderly, while Model IV showed the adjusted odds ratio for QoL after accounting for all selected independent variables associated with injuries. The statistical package STATA for Windows version 14.1^53^ was used for all statistical analyses. The proper individual-level sampling weights were used to make the results representative.

## Results

### Sociodemographic characteristics of older people aged 60 and above in India

Table 1 shows that around one-third (30%) of the older persons were aged 60–64 years, followed by 65–69 years (29%). The proportion of females was 53% of the total older population. Approximately 71% of older people resided in rural areas. Over half (56.5%) of the older people were illiterate, and only 14% of older people had more than ten years of schooling. Over two-thirds (62%) of older people were married, and 36% were widowed. Majority (82%) of older people were Hindus. Most older people were from the Southern region (28%), followed by the Eastern region (22%). Regarding the caste, 45% belonged to other backward classes (OBCs) and 19% to scheduled caste, and only 8% were from scheduled tribes.

**Table 1:** Sociodemographic characteristics of older people aged 60 and above years, India, LASI, (2017-18).

Characteristics	Sample (*n*)	(%)
**Age group**		
60–64 Years	9407	29.9
65–69 Years	9003	28.6
70–74 Years	5898	18.8
75 + Years	7156	22.7
**Sex**		
Male	14,931	47.5
Female	16,533	52.5
**Place of residence**		
Rural	22,196	70.5
Urban	9268	29.5
**Level of education**		
Illiterate (0 year)	17,782	56.5
Less than 5 years	5507	17.5
5–9 years completed	3705	11.8
10 years or more	4469	14.2
**Marital Status**		
Currently married	19,391	61.6
Widowed	11,389	36.2
Others	684	2.2
**Region**		
North	3960	12.6
Central	5642	17.9
East	6802	21.6
North-east	334	1.1
West	5997	19.1
South	8730	27.8
**Caste**		
Scheduled Caste	5947	18.9
Scheduled Tribe	2555	8.1
Other Backward Classes	14,227	45.3
Others	8678	27.6
**Living arrangements**		
Living alone	1787	5.7
Living with spouse	19,176	60.9
Living with other but not spouse	10,501	33.4
**Work Status**		
Currently working	9679	30.8
Ever worked but not working currently	13,469	42.8
Never Worked	8308	26.4
**MPCE wealth quintile**		
Poorest	6829	21.7
Poorer	6831	21.7
Middle	6590	21.0
Richer	6038	19.2
Richest	5175	16.5
**Multimorbidity**		
No morbidity	14,626	46.6
One morbidity	9202	29.3
2 + morbidity	7545	24.1
**Falls**		
No fall	26,235	83.4
Minor fall	1245	4.0
Major fall	3984	12.7
**Injuries**		
No injury	27,212	86.5
Injury	4251	13.5
**Factors of HRQOL**		
**Self-care Health (ADL)**		
No	2647	8.5
Yes	28,689	91.5
**Mobility**		
No	20,459	65.3
Yes	10,877	34.7
**Activities (IADL)**		
No	6984	22.3
Yes	24,311	77.7
**Pain**		
No	18,942	60.4
Yes	12,413	39.6
**Depression CES**		
No	21,211	69.8
Yes	9178	30.2
**Quality of Life (QoL) (0- 0.79)**		
Low QoL	12,003	39.6
Medium QoL	9192	30.3
High QoL	9145	30.1
**Total**	*N* = **31**,**464**	**100.0**

Regarding health behaviours, 60% of the older people never smoked and 85% of older people never consumed alcohol. Over two-thirds (61%) of the older people lived with a spouse, and one-third (33%) lived with others. Furthermore, 31% of the older people were currently working. Regarding self-rated health status, 45% of older people rated their health as fair, and 24% rated their health as poor. About 22% of older people were from poorer/poorest wealth status, 21% to the middle, 19% to the richer, and 16.5% to the richest. In the case of multimorbidity, 29% of older people had only one disease, and 24% had multimorbidity. Over three-fourths (83%) of the older people reported no fall, and 13% reported a major fall. About 14% of older people reported injuries. According to HRQOL factors, such as ADLs (self-care), mobility, IADLs (usual activities), pain, and depression, majority of older people (92%) reported self-care, more than two-thirds (65%) reported no mobility, 22% reported could not do activities, and 40% reported pain. The majority of the older people (70%) were not depressed. QoL measures showed that about 40% of older people had low QoL, and 30% each had a medium and high QoL.

### The sociodemographic differential in falls and injuries among older people in India

Results of Table 2 display that prevalence of falls and injuries tend to increase with age. Serologically, females had higher prevalence of falls (14.4%) than males (10.7%) of whom reported major falls, and they also had higher prevalence of injuries (15.6%). East region people had a higher major fall (17.4%) followed by Central region people (14%) who had a major fall. Similarly, East (19.5%) and Central (14.7%) region people had higher injuries than other region people. While lowest major falls (4.9%) and injuries (6,4%) were found in people from the North-east region. For the type of wealth quintile, the major falls reported (16.9%) were the richest, but the minor falls were low (2.9%), and along with that (15.7%) was the injuries. Furthermore, regarding the number of chronic diseases reported, major falls (16.6%) was two kinds of morbidity, and the reported (16.5%) was injuries.

**Table 2:** Prevalence of falls and injuries with background characteristics among older people aged 60 and above years, India, (LASI),2017-18.

Characteristics	Falls				Injuries		
	No fall	Minor fall	Major fall	*p*-value	No Injury	Injury	*p*-value
**Age group**							
60–64 Years	84.8	3.5	11.7	< 0.001	88.1	11.9	< 0.001
65–69 Years	84.1	3.5	12.4		87.3	12.7	
70–74 Years	82.4	4.4	13.2		85.0	15.0	
75 + Years	81.5	4.7	13.8		84.6	15.4	
**Sex**							
Male	86.1	3.2	10.7	< 0.001	88.8	11.3	< 0.001
Female	80.9	4.6	14.4		84.4	15.6	
**Place of residence**							
Rural	82.7	4.0	13.3	< 0.001	85.8	14.2	< 0.001
Urban	84.9	4.0	11.1		88.1	11.9	
**Level of education**							
Illiterate (0 year)	82.8	4.3	12.9	< 0.001	86.0	14.0	< 0.001
Less than 5 years	81.5	4.9	13.6		85.1	14.9	
5–9 years completed	85.6	3.0	11.5		88.1	11.9	
10 years or more	86.2	2.3	11.5		88.8	11.2	
**Marital status**							
Currently married	85.4	3.2	11.4	< 0.001	88.1	11.9	< 0.001
Widowed	79.7	5.4	15.0		83.5	16.5	
Others	89.4	1.8	8.8		92.1	8.0	
**Region**							
North	88.0	2.8	9.2	< 0.001	91.1	8.9	< 0.001
Central	82.1	3.9	14.0		85.3	14.7	
East	77.5	5.2	17.4		80.5	19.5	
North-east	92.5	2.6	4.9		93.6	6.4	
West	83.8	4.2	11.9		86.5	13.5	
South	86.0	3.4	10.5		89.6	10.4	
**Caste**							
Scheduled Caste	80.8	4.7	14.6	< 0.001	85.2	14.8	< 0.001
Scheduled Tribe	87.0	3.5	9.5		88.6	11.4	
Other Backward Classes	84.2	3.9	11.9		86.6	13.4	
Others	82.7	3.7	13.6		86.6	13.4	
**Living arrangements**							
Living alone	80.6	6.5	12.9	< 0.001	84.2	15.8	< 0.001
Living with spouse	85.4	3.2	11.4		88.1	12.0	
Living with other but not spouse	80.2	4.9	14.9		84.0	16.0	
**Work status**							
Currently working	84.9	3.1	12.0	< 0.001	88.2	11.8	< 0.001
Ever worked but not currently	83.4	4.4	12.3		86.5	13.5	
Never Worked	81.6	4.3	14.1		84.6	15.4	
**MPCE wealth quintile**							
Poorest	85.0	4.5	10.5	0.001	87.4	12.6	0.055
Poorer	83.5	4.0	12.5		87.1	12.9	
Middle	83.5	4.0	12.5		86.5	13.5	
Richer	84.0	4.2	11.9		86.7	13.3	
Richest	80.3	2.9	16.9		84.3	15.7	
**Multimorbidity**							
No morbidity	85.7	3.5	10.8	< 0.001	88.1	11.9	< 0.001
One morbidity	82.9	4.5	12.7		86.2	13.8	
2 + morbidity	79.2	4.2	16.6		83.5	16.5	
**Total**	**83.4**	**4.0**	**12.7**		**86.5**	**13.5**	

Note: ***p < 0.001, **p < 0.01 and *p < 0.05.

### Distribution of falls and injuries by factors associated with health-related QoL among older people

The results of Table 3 specify that those elderly who could not do self-care (ADLs) by themselves found higher percentages of elderly with minor falls (5.8%), major falls (21.7%), and injuries (22.6%). After that, it was observed that those elderly who could not do their usual activities (IADLs) by themselves had higher percentages of older people with minor falls (5.1%), major falls (17.3%), and injuries (18.4%). Results showed that older people with a high QoL reported the lowest prevalence of minor falls (2.6%), major falls (7.5%), and injuries (7.9%). The results showed that as the QoL of the elderly increased, the level of minor falls, major falls, and injuries declined.

**Table 3:** Prevalence of fall and injuries by various factors associated with Health-Related quality of life among the older people aged 60 years and above in India (LASI, 2017-18).

Characteristics	Falls				Injuries		
	No falls	Minor falls	Major falls	*p*-value	No Injury	Injury	*p*-value
**Factors of HRQOL**							
**ADL**							
No	72.5	5.8	21.7	< 0.001	77.4	22.6	< 0.001
Yes	84.3	3.8	11.9		87.2	12.8	
**Mobility**							
No	86.0	3.6	10.5	< 0.001	88.6	11.4	< 0.001
Yes	78.2	4.8	17.0		82.3	17.7	
**IADL**							
No	77.7	5.1	17.3	< 0.001	81.7	18.4	< 0.001
Yes	84.9	3.7	11.5		87.8	12.2	
**Pain**							
No	87.1	3.6	9.4	< 0.001	89.4	10.6	< 0.001
Yes	77.5	4.6	17.9		81.8	18.2	
**Depression CES-D**							
No	84.4	3.5	12.1	< 0.001	87.4	12.6	< 0.001
Yes	80.8	4.9	14.2		84.3	15.7	
**Quality of Life (0.5–0.79)**							
Low QoL	78.4	5.1	16.5	< 0.001	82.5	17.5	< 0.001
Medium QoL	83.1	3.7	13.2		86.1	13.9	
High QoL	90.0	2.6	7.5		92.1	7.9	
**Total**	**83.4**	**4.0**	**12.7**		**86.5**	**13.5**	

Note: ***p < 0.001, **p < 0.01 and *p < 0.05.

### Distribution of QoL with sociodemographic characteristics of older people

Results of Table 4 indicate that all characteristics of older people were found to be statistically significant. The results reveal that people aged 75 + years had the highest percentage with low QoL, and 33% and 37% of older people aged 60–64 had a higher percentage of medium and high QoL, respectively. In the case of sex of the elderly, it was observed that the highest percentage (47%) of women had low QoL, whereas about 31% of men had a low QoL, and 32% and 37% of males had medium and high levels of QoL, respectively, whereas about 29% and 24% of females had medium and high levels of QoL. The results showed that people with higher levels of education had higher percentage of medium and high QoL. More than 44% of people with more than 10 years of level of education had higher percentage of QoL About 35% of older adults with current marital status had high level of QoL Region showed an interesting result, people from the North region (38%) had high QoL, people from North-east region (35%) had medium level of QoL and east region (46%) people had the lowest level of low QoL. Moreover, the results of living arrangements show that the majority (49.6%) of elderly living with others but not spouses or children had a low QoL, whereas 32% and around 35% of elderly living with spouses had medium and high QoL, respectively. Those women who had reported major falls (51.2%) and minor falls (51.1%) were found to have the lowest QoL, while older people with major falls had the highest level of medium QoL, and those elderly who reported no falls were found to have the highest level (32.5%) of high QoL. At the end of this result, it was found that older people with no morbidity had the highest level (37%) of high QoL, whereas older people with one morbidity had the highest level (32%) of medium QoL, and women with 2 + morbidity had the lowest level (52%) of low QoL.

**Table 4:** Distribution of quality of life with background characteristics among older people aged 60 years and above, India, LASI, 2017-18.

Characteristics	Quality of life (QoL)			
	Low QoL	Medium QoL	High QoL	*p*-value
**Age group**				
60–64 Years	30.2	33.1	36.8	< 0.001
65–69 Years	35.3	32.1	32.6	
70–74 Years	42.6	30.5	26.9	
75 + Years	55.7	23.9	20.4	
**Sex**				
Male	30.8	32.1	37.2	< 0.001
Female	47.4	28.7	23.8	
**Place of residence**				
Rural	41.4	29.5	29.1	< 0.001
Urban	35.1	32.3	32.6	
**Level of education**				
Illiterate (0 year)	46.4	27.6	25.9	< 0.001
Less than 5 years	37.3	33.6	29.1	
5–9 years completed	32.4	32.9	34.8	
10 years or more	20.9	34.7	44.5	
**Marital status**				
Currently married	33.3	32.0	34.7	< 0.001
Widowed	50.3	27.6	22.1	
Others	39.7	27.0	33.2	
**Region**				
North	34.0	27.9	38.1	< 0.001
Central	38.7	33.2	28.1	
East	46.1	27.2	26.8	
North-east	28.3	35.4	36.3	
West	40.4	32.6	27.0	
South	37.4	30.3	32.3	
**Caste**				
Scheduled Caste	41.7	28.9	29.4	< 0.001
Scheduled Tribe	36.9	32.8	30.3	
Other Backward Classes	39.9	30.9	29.3	
Others	38.4	29.6	32.0	
**Living arrangements**				
Living alone	49.0	30.9	20.2	< 0.001
Living with spouse	33.2	32.0	34.8	
Living with others but not spouse	49.6	27.1	23.3	
**Falls**				
No falls	37.2	30.2	32.5	< 0.001
Minor falls	51.1	28.9	20.0	
Major falls	51.2	31.2	17.6	
**Injuries**				
No Injury	37.7	30.2	32.1	< 0.001
Injury	51.2	31.2	17.6	
**Work status**				
Currently working	24.9	35.3	39.8	< 0.001
Ever worked but not currently working	46.2	27.5	26.4	
Never Worked	46.5	28.8	24.7	
**MPCE**				
Poorest	42.8	30.6	26.6	< 0.001
Poorer	39.7	30.1	30.2	
Middle	39.2	28.3	32.5	
Richer	38.6	29.6	31.8	
Richest	36.7	33.6	29.7	
**Multimorbidity**				
No morbidity	32.6	30.8	36.7	< 0.001
One morbidity	41.0	31.8	27.2	
2 + morbidity	51.7	27.6	20.8	
**Total**	**39.6**	**30.3**	**30.1**	

Note: ***p < 0.001, **p < 0.01 and *p < 0.05.

### Association of falls and injuries with QoL among older people aged 60 and above

Table 5 results for Models I and III showed the unadjusted odds ratio for falls and injuries, respectively. The Model, I result for falls showed that older people with minor falls were found 45% less likely to have higher QoL as compared to older people with no falls, and older people with major falls were found to be 47% less likely to have higher QoL as compared to those older people with no falls. Afterward, after controlling other sociodemographic and health factors, Model II results showed that older people with minor falls were 30% less likely to have higher QoL than older people with no falls. Furthermore, older people with major falls were 39% less likely to have higher QoL.

**Table 5:** Association between health-related quality of life (HRQOL)^#^ with fall and injuries among older people aged 60 and above years, India, LASI (2017-18).

Characteristics	Model I	Model II	Model III	Model IV
	Unadjusted OR [95% CI]	Adjusted OR [95% CI]	Unadjusted OR [95% CI]	Adjusted OR [95% CI]
**Falls**				
No fall (Ref.)				
Minor fall	0.55***(0.549 0.551)	0.70***(0.698 0.701)		
Major fall	0.53***(0.527 0.528)	0.61***(0.614 0.615)		
**Injuries**				
No injury (Ref.)				
Injury			0.54***(0.538 0.539)	0.65***(0.65 0.651)
**Age group**				
60–64 Years (Ref.)				
65–69 Years		0.91***(0.909 0.91)		0.91***(0.909 0.911)
70–74 Years		0.76***(0.756 0.757)		0.76***(0.756 0.757)
75 + Years		0.51***(0.505 0.506)		0.51***(0.507 0.508)
**Sex**				
Male (Ref.)				
Female		0.69***(0.686 0.688)		0.68***(0.681 0.683)
**Place of residence**				
Rural (Ref.)				
Urban		1.25***(1.248 1.25)		1.25***(1.248 1.25)
**Level of education**				
Illiterate (0 year) (Ref.)				
Less than 5 years		1.26***(1.255 1.258)		1.26***(1.255 1.257)
5–9 years completed		1.44***(1.437 1.441)		1.44***(1.437 1.44)
10 years or more		2.44***(2.433 2.439)		2.44***(2.437 2.443)
**Marital Status**				
Currently married (Ref.)				
Widowed		0.89***(0.884 0.892)		0.90***(0.897 0.905)
Others		0.90***(0.892 0.901)		0.91***(0.906 0.915)
**Region**				
North (Ref.)				
Central		0.62***(0.618 0.62)		0.62***(0.615 0.617)
East		0.54***(0.544 0.545)		0.54***(0.543 0.544)
North-east		0.89***(0.885 0.891)		0.90***(0.892 0.899)
West		0.60***(0.601 0.603)		0.60***(0.6 0.602)
South		0.81***(0.806 0.808)		0.81***(0.804 0.806)
**Caste**				
Scheduled Caste (Ref.)				
Scheduled Tribe		1.00***(1.004 1.007)		1.02***(1.014 1.017)
Other Backward Classes		1.00***(0.995 0.997)		1.01***(1.006 1.008)
Others		0.98***(0.983 0.985)		0.99***(0.984 0.986)
**Living arrangements**				
Living alone (Ref.)				
Living with spouse		1.17***(1.168 1.179)		1.19***(1.186 1.197)
Living with other but not spouse		1.09***(1.09 1.094)		1.09***(1.085 1.089)
**Work Status**				
Currently working (Ref.)				
Ever worked but not working currently		0.59***(0.586 0.587)		0.59***(0.589 0.59)
Never Worked		0.72***(0.716 0.717)		0.73***(0.724 0.725)
**MPCE wealth quintile**				
Poorest (Ref.)				
Poorer		1.16***(1.158 1.16)		1.15***(1.149 1.152)
Middle		1.20***(1.198 1.2)		1.20***(1.195 1.198)
Richer		1.19***(1.185 1.188)		1.18***(1.182 1.185)
Richest		1.18***(1.179 1.182)		1.17***(1.164 1.167)
**Multimorbidity**				
No morbidity (Ref.)				
One morbidity		0.64***(0.639 0.64)		0.64***(0.637 0.638)
2 + morbidity		0.38***(0.383 0.383)		0.38***(0.379 0.38)
/cut1	−0.54(−0.536 −0.535)	−1.54(−1.544 −1.534)	−0.51(−0.51 −0.51)	−1.51(−1.51 −1.5)
/cut2	0.75(0.744 0.745)	−0.10(−0.103 −0.093)	0.76(0.76 0.76)	−0.07(−0.07 −0.07)

Note: Ref: Reference categories, OR = Odds Ratio; CI = Class Interval; ***p < 0.001, **p < 0.01 and *p < 0.05.

# indicate the outcome variable of interest.

Following that, in model III result, which is a crude result for injury, revealed that older people with injuries were 46% less likely to have higher QoL than those without injuries. In addition, after controlling for all other variables, Model IV revealed that older people with injuries were 35% less likely to have higher QoL than those without injuries.

The results of the controlling factors were also statistically significant for falls and injuries. The results of models II and model IV showed that as older people age increases, the chances of having higher QoL decreases significantly among those older people who have fallen and been injured. Regarding the sex of the elderly, the results showed that older females were 31% and 32% less likely to have higher QoL than elderly males. Older people who lived in urban areas and had good education were more likely to be able to lead a good life. The results showed that urban people were 1.25 times more likely to have higher QoL than rural people. The education level showed that older people with more than 10 years of schooling were 2.44 times more likely to have higher QoL than illiterate people. The region-wise results suggest that people from the east region were 46% less likely to have falls and injuries than the north region people whereas south region people were only 19% less likely to have falls and injuries than the north region. Those living with a spouse had a 1.17 times higher likelihood of QoL than older people living alone. Furthermore, as older people’s wealth status increases, the chances of having higher QoL rise significantly. The higher the morbidity, the lower the possibility of having higher QoL. Results for cut1 represents the threshold boundary between the first and second categories (e.g., low vs. medium/high), and cut2 represent the threshold boundary between the second and third categories (e.g., low/medium vs. high).

## Discussion

This study examined the linkage of quality of life (QoL) with falls, and injuries among older people in India. This study identified the key factors associated with falls and injuries among older people in India. The findings suggest that sex, living arrangement, place of residence, work status, multimorbidity, and QoL were significantly related to falls and injuries. The study’s findings show that more than half of the respondents were older women. More than 70% of older people were residing in rural areas and more than 50% of older people were illiterate, and more than two-thirds of older people lived with their spouses. Over 50% of older people were suffering from either one or multimorbidity; 29% with single morbidity, and 24% with multimorbidity. About 13% had experienced falls and 14% experienced injuries.

There are five stages in the human life cycle: childhood, adolescence, youth, adulthood, and old age. Old age is the last stage of human life. At this stage, the health is naturally more declines. So, this research aimed to examine the relationship between QoL, falls, and injuries among older people in India. QoL has become the most critical issue in recent times. Several background characteristics directly impact the QoL^54,55^. With increasing age of people, the chances of falls and injuries also increases, causing the lower QoL. This study findings showed that older people with major falls were 39% less likely to have a better QoL than those without falls. Similar studies from Taiwan, Norway, and Germany indicate that due to falls, older people lose their QoL^56,57^ and some studies from India report the same pattern^58,59^. Besides these, one of the medical studies from Soudi Arabia also revealed that falls was the negative contributors for the QoL of the older persons^60^. Likewise, in the case of injuries, findings also indicate that older people with injuries were 35% less likely to have a better QoL than older people with no injuries; some similar studies from Korea, North India, and the United Kingdom suggest that the QoL of older people diminished and make them more vulnerable in the society elderly to those elderly with injuries^22,61,62^. Together, these findings highlight the significant negative impact of falls and injuries on the QoL of older people in India.

Furthermore, older people aged 75 and above who experience of falls and injuries were 49% less likely to have a better QoL than those 60–64 years old in India. This finding showed that with increasing age, different health issues arise, such as illness, diseases, etc. which caused low QoL in later life^63,64^. Female respondents were more likely to report poorer QoL, reflecting gender disparities in Indian society^65^. Urban residents had a 25% higher likelihood of better QoL than rural residents. In urban areas, people can efficiently access services, facilities, and basic amenities^66^. In contrast, community-based studies from urban slum and urban Puducherry in India suggest that lack of physical activity and burden of multimorbidity reflect in their life as poor QoL^67,68^.

Higher education levels were linked to a better QoL, with those educated for over ten years experiencing 2.4 times higher QoL than illiterate individuals. Education raises awareness and empowers both individuals and society, contributing to improved well-being^69^. Married older people had a better QoL than widowed or single individuals, possibly due to mutual support in later life^70^ Economic status also played a role: those with higher monthly per capita expenditure enjoyed better QoL, reflecting greater access to resources and services^41–44,48,66,72^.

Morbidity remains a key factor directly reducing QoL^71^. Older people with one morbidity were 36% less likely to have better QoL, and those with two or more were 62% less likely than those without any morbidity. As morbidities increase, they strain physical, mental, and financial well-being, leading to a decline in QoL^72^. These findings align with findings from other Indian studies, which reported that higher age, female gender, rural residence, widowerhood, lower education level, Scheduled Tribe, Muslims, rich household wealth status, east and south region, and multimorbidity condition were associated with poorer HRQOL^47^. The recent studies from rural areas of India indicates that age, lower economic status, divorce, multimorbidity conditions, female gender, and physically inactive are linked to poor QoL^73–76^.

A Key limitation of the study is its cross-sectional design, which restrict the causal inference. Additionally, reliance on self-reported data for health outcomes may introduce reporting bias. Despite these limitations, the study addresses a critical research gap in understanding the QoL-related to falls and injuries in India and offers findings that are generalizable at the national level. India’s older adults should be provided with improved QoL through nationwide fall prevention programs, enhanced healthcare access, safe living environments, and strengthened social support systems. Awareness campaigns on fall risks and prevention strategies should be promoted, with regular monitoring for effectiveness.

## Conclusions

The findings of this study revealed that various factors significantly influence the QoL among older adults concerning falls and injuries. Major falls were associated with a notable decline in QoL, particularly among those with advancing age, unmarried status, and multiple morbidities, all of which showed a significant negative correlation. In contrast, higher education, better living arrangements, urban residence, and greater monthly per capita expenditure were positively associated with improved QoL. These results highlight the importance of identifying and addressing factors that adversely affect QoL, enabling older adults to lead healthier and more fulfilling lives. Additionally, adopting safety measures and enhancing social participation were found to support better QoL outcomes. To reduce fall-related risks and improve overall well-being, both older individuals and their families should be educated on preventive strategies, and targeted interventions should be implemented.

## Supplementary Information

Below is the link to the electronic supplementary material.

Supplementary Material 1

## Data Availability

This study utilizes a secondary data source of data that is freely available by using the given link by registration and login to request the data set for use. [https://www.iipsdata.ac.in/datacatalog\_detail/5](https:/www.iipsdata.ac.in/datacatalog_detail/5). The data request form can be easily downloaded by given link ([*LASI Wave 1 Data Request form (iipsindia.ac.in)](https:/iipsindia.ac.in/sites/default/files/LASI_DataRequestForm_0.pdf)).

## Abbreviations

HRQOL: Health-related Quality of Life
QoL: Quality of Life
MPCE: Mean Per Capita Expenditure
ADL: Activities of Daily Living
IADL: Instrumental Activities of Daily Living
CESD-scale: Centre for Epidemiological Studies-Depression-scale
EQ-5D: Euro-QOL five dimensions questionnaire
VAS: Visual Analog Scale
MPCE: Monthly Per Capita Expenditure
