# Supplementary material for: Linkages of quality of life with falls and injuries among older people in India
Source: Sci Rep. 2025 Nov 24;15:45240. doi: 10.1038/s41598-025-29486-1 (PMC12749978; doi:10.1038/s41598-025-29486-1)
Supplement: Supplementary file 1 — Supplementary Material 1 [file 41598_2025_29486_MOESM1_ESM.docx]

**Appendix-1**

**Creation of HRQOL index by using PCA**.

There are five dimensions of HRQOL have been used to create the index of HRQOL

1. **The first dimension of EQ-5D measurement is defined as *mobility*.**

In the LASI, a total of nine questions were asked related to measuring mobility conditions.

| **Sr. No.** | **Variable available in LASI** | **Mobility questions** |
| --- | --- | --- |
| 1 | ht303 | Difficulty in walking 100 yards |
| 2 | ht304 | Difficulty in sitting for 2 h or more |
| 3 | ht305 | Difficulty in getting up from a chair after sitting for a long period |
| 4 | ht306 | Difficulty in climbing one flight of stairs without resting |
| 5 | ht307 | Difficulty in stooping, kneeling, or crouching |
| 6 | ht308 | Difficulty in reaching or extending arms above shoulder level (either arm) |
| 7 | ht309 | Difficulty in pulling or pushing large objects |
| 8 | ht310 | Difficulty to lift or carry weights over 5 kilos |
| 9 | ht311 | Difficulty in picking up a coin from a table |

All mobility conditions with yes were recorded as 1 (yes “having difficulty”) and all no combined in 0 (no “no difficulty”).

1. **The second dimension in the EQ-5D measurement of *Self-care* measures**

In LASI, a total of six questions related to self-care were asked to the respondents.

| **Sr. No.** | **Variable available in LASI** | **Self-care (ADL)** |
| --- | --- | --- |
| 1 | ht401 | Difficulty with dressing, including putting on chappal and shoes |
| 2 | ht402 | Difficulty with walking across a room |
| 3 | ht403 | Difficulty with bathing |
| 4 | ht404 | Difficulty with eating |
| 5 | ht405 | Difficulty with getting in or out of bed |
| 6 | ht406 | Difficulty with using the toilet, including getting up and down |

All self-care conditions were recorded as (1 = yes “having difficulty” and 0 = no “no difficulty”), and a total score of 0–6 was generated in the self-care dimension. Furthermore, converted into binary with a score 0/2 =1 “have a problem in self-care” and 3/6=0 “have no problem in self-care.

1. **The second dimension in the EQ-5D measurement of *usual activities (IADL)* measures**

In LASI, a total of seven questions were asked related to usual activities (IADL)

| **Sr. No.** | **Variable available in LASI** | **Usual activities (IADL)** |
| --- | --- | --- |
| 1 | ht407 | Difficulty with preparing a hot meal |
| 2 | ht408 | Difficulty with shopping for groceries |
| 3 | ht409 | Difficulty with making telephone calls |
| 4 | ht410 | Difficulty with taking medications |
| 5 | ht411 | Difficulty with work around the house or garden |
| 6 | ht412 | Difficulty with managing money, such as paying bills and keeping track of expenses |
| 7 | ht413 | Difficulties with getting around or finding addresses in unfamiliar places |

All usual activities variable conditions were recorded as (1=yes “have difficulty” and 0= no “no difficulty”) and a total score of 0-7 was generated in the usual activities dimension. Furthermore, converted into binary with a score 0/3 =1 “have a problem in usual activities” and 4/7=0 “have no problem in usual activities.

1. **The fourth dimension of the EQ-5D of *pain and discomfort* measures**

In LASI, a question was asked if patients had trouble with pain.

The answer was recorded as (1 = yes “having pain” and 0 = no “no pain”).

1. **The fifth dimension in the EQ-5D measurement of *Anxiety & Depression*,**

In LASI, questions related to measuring depression symptoms by using the CES_D (Center for Epidemiological Studies-Depression) scale55. The questions were included to measure depression in the study as Trouble concentrating, feeling depressed, feeling tired or low in energy, feeling afraid of something, feeling overall satisfied, feeling alone, bothered by things, everything you did was an effort, not Feeling happy, Hopeful about the future, and feel overall satisfied.

| **Sr. No.** | **Variable available in LASI** | **CES-D (Depression)** |
| --- | --- | --- |
| 1 | fs701 | Trouble concentrating |
| 2 | fs702 | Feel depressed |
| 3 | fs703 | Feel tired or low in energy |
| 4 | fs704 | Feel afraid of something |
| 5 | fs705 | Feel overall satisfied |
| 6 | fs706 | Feel alone |
| 7 | fs707 | Bothered by things |
| 8 | fs708 | Everything you did was an effort |
| 9 | fs709 | Hopeful about the future |
| 10 | fs710 | Feel happy |

In the first seven questions, the answer was recorded as 1 = yes if the person was feeling more than three days in a week; otherwise, 0 = no recorded, but the last three questions were recorded as 1 = yes if feeling more than three days in a week otherwise as recorded as 0. A total score of 0–10 was generated for depression and anxiety dimensions. Furthermore, converted into binary with score 0/3 =1 “no depression” and 4/10=0 “have depression”

**Creation of HRQOL index by using principal component analysis (PCA)**

After converting all dimensions of HRQOL into the binary nature. PCA command has been used with the dimension of the HRQOL.

***The command used to create the index:***

pca mobility2 selfcare2 activities2 pain2 depression_CES


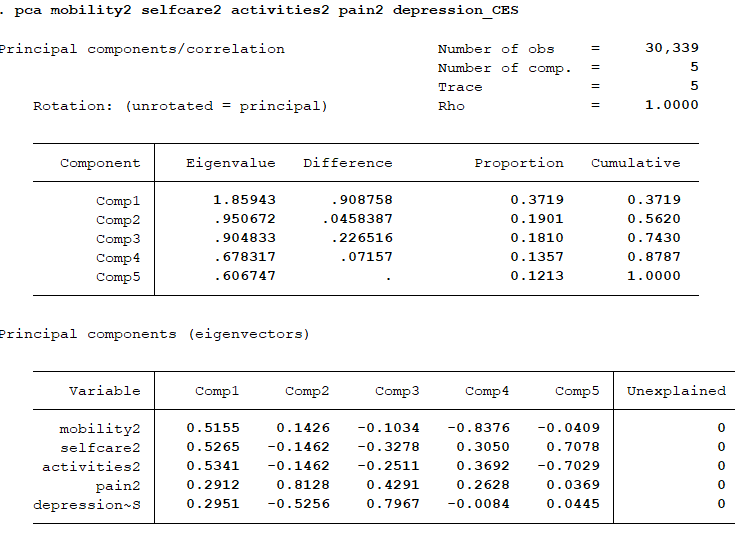


This output suggests that component one has a higher eigenvalue and this explains more than 37% of the variation.

**Scree plot:**

The scree plot also suggests that one component eigenvalue is greater than one and the second is close to one. Which combination explains more than 56% of the variation in the model.

**predict comp1, score**


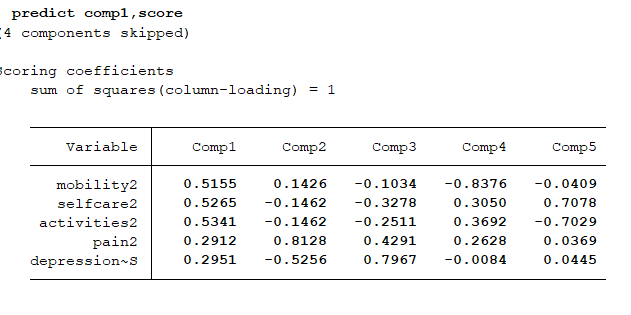


egen min_comp1=min(comp1)

**generation of index**

g index = comp1+abs(min_comp1)

**ta index: index of quality of life**

| **Index (HRQOL)** | **Freq.** | **Percent** | **Cum.** |
| --- | --- | --- | --- |
| 0 | 515 | 1.7 | 1.7 |
| 0.593298 | 281 | 0.93 | 2.62 |
| 0.657224 | 327 | 1.08 | 3.7 |
| 1.103395 | 45 | 0.15 | 3.85 |
| 1.250521 | 290 | 0.96 | 4.81 |
| 1.386635 | 127 | 0.42 | 5.22 |
| 1.696693 | 28 | 0.09 | 5.32 |
| 1.760618 | 44 | 0.15 | 5.46 |
| 1.979933 | 57 | 0.19 | 5.65 |
| 2.043859 | 177 | 0.58 | 6.23 |
| 2.051244 | 544 | 1.79 | 8.03 |
| 2.353916 | 36 | 0.12 | 8.14 |
| 2.49003 | 27 | 0.09 | 8.23 |
| 2.637156 | 99 | 0.33 | 8.56 |
| 2.644542 | 337 | 1.11 | 9.67 |
| 2.708467 | 679 | 2.24 | 11.91 |
| 3.083328 | 22 | 0.07 | 11.98 |
| 3.147253 | 47 | 0.15 | 12.14 |
| 3.154638 | 312 | 1.03 | 13.16 |
| 3.301765 | 561 | 1.85 | 15.01 |
| 3.437879 | 950 | 3.13 | 18.14 |
| 3.740551 | 29 | 0.1 | 18.24 |
| 3.747936 | 337 | 1.11 | 19.35 |
| 3.811862 | 495 | 1.63 | 20.98 |
| 4.031177 | 817 | 2.69 | 23.68 |
| 4.095102 | 2,012 | 6.63 | 30.31 |
| 4.40516 | 666 | 2.2 | 32.5 |
| 4.541274 | 1,331 | 4.39 | 36.89 |
| 4.6884 | 1,990 | 6.56 | 43.45 |
| 5.134572 | 2,762 | 9.1 | 52.55 |
| 5.198497 | 4,635 | 15.28 | 67.83 |
| 5.791795 | 9,760 | 32.17 | 100 |
| **Total** | **30,339** | **100** |  |

**Range of quality of life (QoL) is 0 to 5.79.**

**Converted into three categories**

xtile hrqol=index, nq(3)

The frequency of health-related quality of life:

ta hrqol[aw=indiaindividualweight]

| **Health related quality of life** | **Freq.** | **Percent** |
| --- | --- | --- |
|  |  |  |
| Low quality of life | 12,002 | 39.6 |
| Medium quality of life | 9,192 | 30.3 |
| High quality of life | 9,145 | 30.1 |
| **Total** | **30,339** | **100** |

The range of QoL is 0 to 5.79 with this cut-off after extile. It is found that the low QoL cut-off is 0 to below 4.6, and for medium QoL cut-off range includes 4.6 to 5.19 and lastly 5.79 is the cut-off of the high QoL in the given study.
